# Effectiveness and cost-effectiveness of home-based postpartum care on neonatal mortality and exclusive breastfeeding practice in low-and-middle-income countries: a systematic review and meta-analysis

**DOI:** 10.1186/s12884-019-2651-6

**Published:** 2019-12-18

**Authors:** Gizachew Tadele Tiruneh, Chalachew Bekele Shiferaw, Alemayehu Worku

**Affiliations:** 1 The Last Ten Kilometers (L10K) Project, JSI Research & Training Institute, Inc., Addis Ababa, Ethiopia; 2 St. Paul Hospital Millennium Medical College, Birhan Health and Demographic Surveillance System, Addis Ababa, Ethiopia; 3 Addis Ababa University School of Public Health, Addis Ababa, Ethiopia

**Keywords:** Cost, Cost-effectiveness, Exclusive breastfeeding, Home visit, Home-based postnatal care, Home-based newborn care, Low-and-middle-income countries, Neonatal mortality, Postpartum home visit

## Abstract

**Background:**

Early postpartum facility discharge negatively impacts mothers’ proper and effective use postnatal care. Cognizant of these facts, home-based postnatal care practices have been promoted to complement facility-based care to reduce neonatal mortality. This systematic review evaluated the effectiveness and cost-effectiveness of home-based postnatal care on exclusive breastfeeding practice and neonatal mortality in low-and-middle-income countries.

**Methods:**

Randomized trials and quasi-experimental studies were searched from electronic databases including PubMed, Popline, Cochrane Central Register of Controlled Trials and National Health Service Economic Evaluation databases. Random-effects meta-analysis model was used to pool the estimates of the outcomes accounting for the variability among studies.

**Results:**

We identified 14 trials implementing intervention packages that included preventive and promotive newborn care services, home-based treatment for sick neonates, and community mobilization activities. The pooled analysis indicates that home-based postpartum care reduced neonatal mortally by 24% (risk ratio 0.76; 95% confidence interval 0.62–0.92; 9 trials; *n* = 93,083; heterogeneity *p* < .01) with no evidence of publication bias (Egger’s test: Coef. = − 1.263; *p* = .130). The subgroup analysis suggested that frequent home visits, home visits by community health workers, and community mobilization efforts with home visits, to had better neonatal survival. Likewise, the odds of mothers who exclusively breastfed from the home visit group were about three times higher than the mothers who were in the routine care group (odds ratio: 2.88; 95% confidence interval: 1.57–5.29; 6 trials; *n* = 20,624 mothers; heterogeneity *p* < .01), with low possibility of publication bias (Coef. = − 7.870; *p* = .164).

According to the World Health Organization’s Choosing Interventions that are Cost-Effective project recommendations, home-based neonatal care strategy was found to be cost-effective.

**Conclusions:**

Home visits and community mobilization activities to promote neonatal care practices by community health workers is associated with reduced neonatal mortality, increased practice of exclusive breastfeeding, and cost-effective in improving newborn health outcomes for low-and-middle-income countries. However, a well-designed evaluation study is required to formulate the optimal package and optimal timing of home visits to standardize home-based postnatal interventions.

## Background

Globally, about three-quarters of neonatal deaths occur during the first week of life, of which about half occur in the home [1]. More than half of all neonatal deaths could be averted with care being provided during the postpartum period (i.e., 30% of all neonatal deaths can be averted with care of small and ill neonates, 12% with care of healthy neonates, and 10% with immediate newborn care) [2]. A wealth of evidence exists on a range of cost-effective life-saving maternal and newborn health (MNH) interventions essential to end preventable maternal and neonatal deaths [2, 3]. But, in low-and-middle-income countries (LMICs), access to evidence-based high impact interventions improving maternal and neonatal mortality is often low [4–6].

Despite the critical importance of the postnatal period to promote optimal essential neonatal care practices as well as to save the lives of neonates [2], many women and their newborns do not have access to health care during the early postnatal period. This puts both the women and their newborns at an increased risk of morbidity and mortality [7]. Furthermore, due to cultural practices, mothers and newborns spend most of the postnatal period in the home regardless of place of birth [8]. Early postpartum discharge from facility is another factor that contributes to the lack of proper postnatal care (PNC) use [9, 10].

Cognizant of these facts, providing home-based postpartum care is crucial to reach mothers and newborns [9]. As a result, home-based postnatal care practices have been promoted to complement facility-based care to improve early postpartum MNH care as well as to reduce neonatal mortality [11, 12].

Evidence in developing countries shows that community-based postpartum interventions are effective in improving MNH service use [3, 12–17]. However, there is limited evidence of the effectiveness as well as cost-effectiveness of home-based postpartum care strategy [18] and it is also an area where there is a critical knowledge gap, globally [19].

Thus, this systematic review evaluated the effectiveness and cost-effectiveness of home-based PNC on exclusive breastfeeding practice and neonatal mortality in LMICs.

## Methods

Researchers determined the review methods in advance and we registered the protocol on the International Prospective Register of Systematic Reviews (PROSPERO) (registration number: CRD42018106006).

### Inclusion and exclusion criteria

Randomized trials and quasi-experimental studies that report effectiveness or cost-effectiveness of postpartum home-based interventions on practice of exclusive breastfeeding and neonatal mortality or economic evaluation studies that compared home-based interventions with routine care were included. Articles written in the English language, with no publication date restriction to get adequate studies and interventions implemented in LMIC were considered for this study.

Citations without abstracts and/or full text, commentaries, letters, duplicate studies and editorials, studies written by different languages other than English were excluded from the review. Additionally, studies were excluded if it, 1) did not report on postpartum home visit, newborn mortality, exclusive breastfeeding, or cost, 2) did not have comparator, and 3) were not done in LMIC setting.

### Search strategy

The research team searched peer-reviewed journal articles from electronic databases including PubMed, Popline, ClinicalTrials.gov, Cochrane Central Register of Controlled Trials and National Health Service (NHS) Economic Evaluation databases. For more articles, reference lists of the initial search were also checked.

First, we identified concepts including 1) effectiveness or cost-effectiveness, 2) postpartum care or neonatal care, and 3) LMICs using the PICO review acronym—Population, Intervention, Comparison, and Outcome. Then for each concept, search terms (including synonyms and MeSH terms) were identified and used in a variety of combinations for these concepts. For database search, combination of the following keywords were used “(postnatal care OR postpartum OR puerper* OR post partum OR post natal OR neonatal OR newborn) AND (home visit OR home care OR facilit* OR hospital care OR health center care OR facility based care OR home care service*) AND (infant mortality OR neonatal mortality) OR (breastfeeding OR exclusive breastfeeding) OR (cost benefit analysis OR cost analysis OR efficien* OR health care cost OR economic evaluation OR cost effectiveness OR impact OR effect*))”. A search strategy for PubMed, Popline, Cochrane Central Register of Controlled Trials and NHS Economic Evaluation databases are included in the additional file (Additional file 1). ClinicalTrials.gov was searched for recently completed trials.

## Definition of terms

### Intervention

Interventions—including counseling; examination and management; and provision of services—provided to women and newborns in the first six weeks after birth at home by health providers or community health workers.

Any home visit innovations, initiatives, approaches, or activities carried out during the postnatal period, with the aim of either providing maternal and newborn health service or influencing maternal and newborn care-seeking behavior and practices, including home-based treatment for illness, community mobilization efforts, and any home-based/community-based neonatal interventions, were considered as home-based postnatal care.

### Comparison

Routine postnatal care provided to mothers who delivered in a health facility on discharge or care provided after discharge within six weeks when the women visited the facility. Usually, in LMICs, PNC is provided on discharge after facility delivery within 6 h and when the women visited the facility for family planning or immunization services after discharge within six weeks.

### Settings

Low-and-Middle-Income Countries are countries with a gross domestic product (GDP) per capita of less than US $4655 as categorized by the World Bank in 2017. The list of countries is included in the additional file (Additional file 1).

### Outcomes

The definitions for the outcome variables— neonatal mortality, exclusive breastfeeding, and cost-effectiveness—are given below.

*Neonatal mortality:* neonates who died within the first 28 days of life.

*Exclusive breastfeeding****:*** women who exclusively breastfed their child as per the age of the neonate determined by each study at the time of the survey.

*Cost-effectiveness*: Cost per effect as measured by the cost per home visit, per life-saved (or death averted), per life-year saved (or years of life lost averted), or per disability-adjusted life-year (DALY) averted.

The cost results reported in non-US dollars (USD) were converted to USD and inflated to 2016 prices [20, 21]. The GDP per capita was used as a benchmark to consider against the cost-effectiveness of the intervention. For this purpose, the 2016 country’s USD prices were used [21]. World Health Organization (WHO) considers strategies and interventions to be cost-effective if the cost per DALY averted is less than three times the GDP per capita and highly cost-effective if less than the GDP per capita [22].

### Selection and management of results

The search returned 1081 records after removing duplicates. Endnote reference manager was used to upload search results and create library of all search results for the purpose. Two review authors (GT and CB) independently screened the titles and abstracts yielded by the search against the inclusion criteria. We obtained full reports for all titles that appear to meet the inclusion criteria. Discrepancies between reviewers regarding the decision of inclusion were resolved through discussions.

During the title and abstract review stage, publications were excluded based on the exclusion criteria. Additionally, at the full-text review stage, publications were excluded if it did not report on our outcome measures and presented secondary rather than primary analysis (in which case the references were checked for more articles for inclusion).

**Fig. 1:**
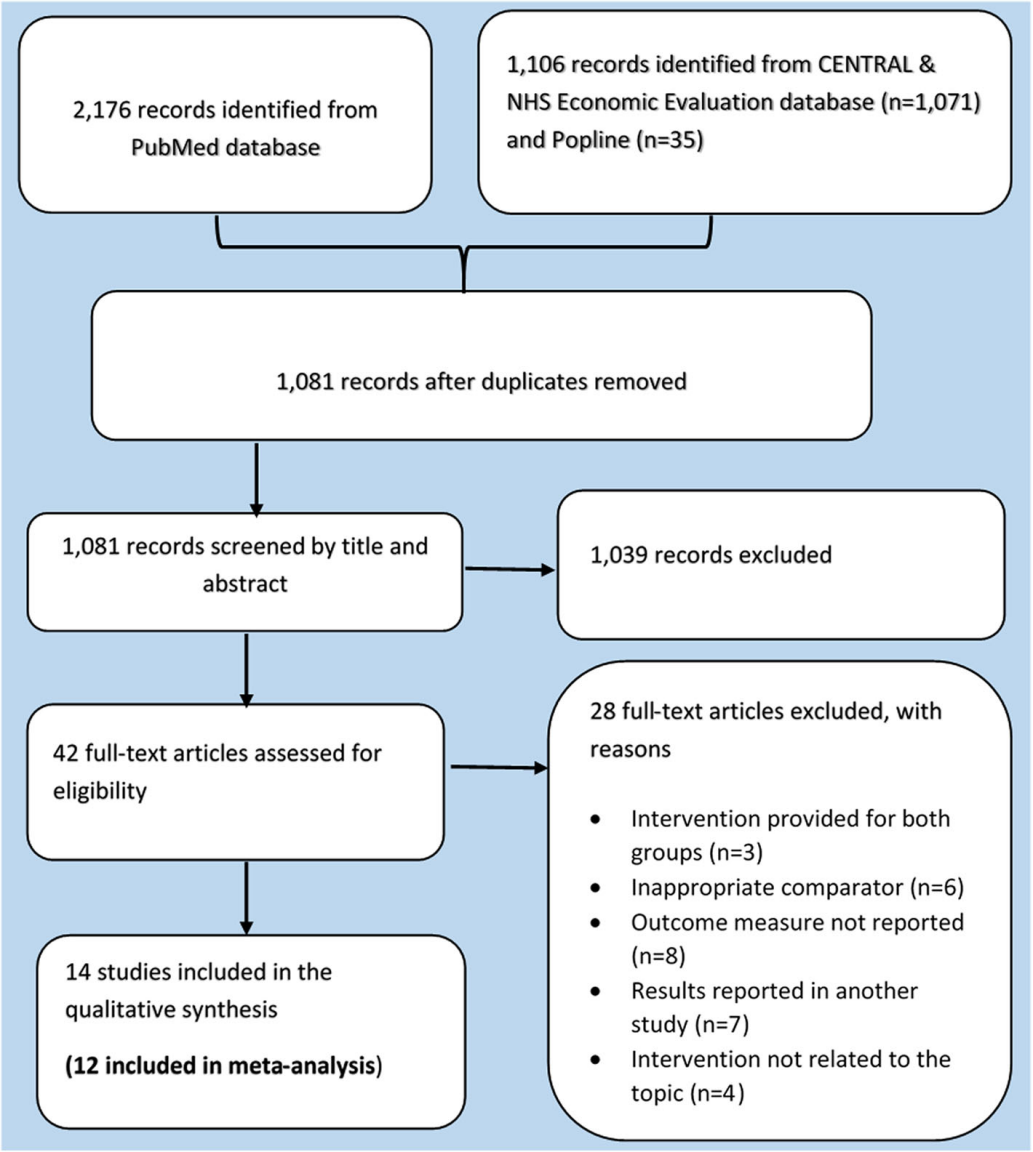
Study flow diagram

Accordingly, 42 full articles were identified from screening titles and abstracts. The final synthesis was based on 14 journal articles. The results of the search and the process of screening and selecting studies for inclusion are illustrated using the study flow diagram seen below (Fig. 1).

### Quality assessment

The risk of bias of each included study was assessed using the Cochrane Collaboration criteria: sequence generation, allocation concealment, blinding of participants and personnel, blinding of outcome assessment, incomplete data, selective reporting and other biases [23]. Through discussion, researchers evaluated the possible risk of bias of each of the six domains from the extracted information and rated as high risk, low risk or unclear, if there was insufficient detail reported in the study, based on the criteria for judging the risk of bias. Graphic representations of risk of bias within and across studies were computed using Review Manager Software v5.3 (RevMan) software [23, 24].

In cluster-randomized trials, allocation concealment was treated as low risk if trials randomized all clusters at once though it lacked concealment of an allocation sequence [23]. In addition, recruitment bias, contamination bias, baseline imbalance, loss of clusters, and lost to follow-up were considered in the analysis of the risk of bias in cluster-randomized trials.

The quality of the evidence pertaining to the outcomes was graded using the Grading of Recommendations Assessment, Development and Evaluation (GRADE) approach based on study limitations, consistency of effect, imprecision of effect estimates, indirectness of evidence and potential publication bias [23]. Researchers jointly evaluated the quality of the evidence for each outcome through discussion and a Summary Findings table was produced using GRADEPro software [25].

Standard reporting checklists—Preferred Reporting Items for Systematic and Meta-Analysis (PRISMA) for systematic review and meta-analysis and International Society for Pharmacoeconomics and Outcomes Research (ISPOR) for the trial-based cost-effectiveness analysis— were followed to establish minimum information that should be included when reporting.

The quality of the trial-based cost-effectiveness studies was assessed based on the core items recommended for conducting economic analyses alongside clinical trials [26]. Each study was given a 1-point score for each item fully met, 0.5 for partially met, and 0 for none. Then, trials that scored 75% or more were categorized as high quality, trials scored between 50 and 74% were ranked as medium, and studies scored less than 50% scores were rated as poor.

### Data extraction

The first reviewer (GT) completed the data extraction form for all studies using RevMan software [23, 24], and the second reviewer (CB) assessed the accuracy of the extracted data. Differences were resolved through discussions.

For those cluster-randomized trials where the analysis did not properly account for cluster design or did not have sufficient information regarding proper accounting of cluster design, we extracted the data after correcting the analysis to reduce the size of each trial to its effective sample size as described in the Cochrane Handbook [23]. The effective sample size of a single intervention group in a cluster-randomized trial is its original sample size divided by the design effect. The design effect is given by.

$$ 1+\left(M-1\right) ICC, $$

where M is the average cluster size and ICC is the intra-cluster correlation coefficient.

We used an estimate of 0.02 ICC derived from the previous trials [27–32].

We identified a trial [33] which included more than two arms. As such, we combined the groups using the methods described in the Cochrane Handbook [23] to analyze them.

### Data synthesis

Descriptive information about the eligible studies was summarized using text and Tables. A narrative synthesis was used to analyze and interpret the findings.

Random-effects meta-analysis model [23, 34] was used to pool the estimates of the outcomes, accounting for the variability among studies using Stata v15 [35]. Both single and multiple level meta-regression analysis was done to identify the independent predictor variables for the outcome of interest.

The results were presented as risk ratio (RR) & odds ratio (OR) with 95% confidence intervals and the estimates of Tau^2^ and I^2^ statistic for heterogeneity. We also investigated the presence of publication and other bias in the extracted data using funnel plot and Stata’s “metabias” command [34, 35].

However, meta-analysis was not appropriate for the cost-effectiveness outcomes as the analytic approaches were different among trials which makes it difficult to combine the outcomes together.

### Assessment of heterogeneity

The *P-*value of the Chi-squared test of heterogeneity and the I^2^ and Tau^2^ statistics were examined for heterogeneity between the trials to judge whether there were any apparent differences in the direction or size of the treatment effect between studies. The thresholds for the presence of heterogeneity among the trials were determined if the value of I^2^ was greater than 30%, and the value of Tau^2^ was greater than zero or the *P-*value of the Chi^2^ test for heterogeneity was greater than 0.1.

### Subgroup analysis

We undertook subgroup analysis to examine the effect of home visit on neonatal mortality varied by 1) number or frequency of PNC visits (< 3 vs. > 3 visits), 2) coverage of PNC visits made to the mother and/or newborn that is proportion of neonates and/mothers receiving a postnatal visit in the intervention areas/clusters) (< 70% vs. > 70%), 3) type of care (preventive interventions vs preventive and curative interventions, 4) type of health worker (health professionals vs. community health workers (CHWs)), 5) intervention components (community mobilization efforts and home visits vs. home visits alone), 6) time of home visits (antepartum and postpartum vs. postpartum only), and 7) publication year (< 2008 vs. > 2008). Likewise, subgroup analysis was done for exclusive breastfeeding outcome which varied by, 1) number or frequency of PNC visits (< 3 vs. > 3 visits), 2) coverage of PNC visits (< 70% vs. > 70%), 3) duration of intervention or follow-up (< 4 vs. > 4 weeks), 4) age at exclusive breastfeeding (neonatal period vs. more than neonatal period), 5) type of health worker (health professionals vs. CHWs), and 6) publication year (< 2008 vs. > 2008).

In subgroup analysis, we presented both random-effects and fixed-effects model estimates to visualize the presence of small-study effects; however, due to substantial heterogeneity, we preferred to discuss the results acquired from random-effects model.

### Sensitivity analysis

We conducted a sensitivity analysis excluding trials that evaluated the effect of home-based follow-up of neonates born at and recruited from hospitals, to determine the pooled estimate of trials with home-based neonatal care which had poor access to health facilities.

## Results

### Description of studies

The characteristics of included studies are given in Table 1 below. Fourteen articles (2 randomized controlled trials (RCT), 11 cluster-randomized trials, and 1 quasi-experimental studies) were included. All included trials were published from 1999 to 2017.

**Table 1:** Characteristics of included studies

Study ID	Design	Setting	Participants	Effective sample size (intervention/control)	Outcomes	Notes
Bang 1999 [38]	Cluster RCT	Gadchiroli district of India which is about 1000 km from the state capital, Mumbai. It is underdeveloped district, with poor infrastructure (poor roads, communications, education, and health services)	Newborns in 39 intervention and 47 control villages (2869 and 3122 newborns, respectively)	1209/1315	Neonatal mortality, stillbirth, perinatal mortality, and cause of neonatal death	
Baqui 2016 [39]	Cluster RCT	Beanibazar, Zakiganj and Kanaighat subdistricts of Sylhet division of Bangladesh	Newborns within the general community of the Sylhet district in rural northeast Bangladesh (9630 and 9852 newborns in the intervention and control groups, respectively)	380/389	Neonatal mortality and cause-specific neonatal mortality	
Bashour 2008 [33]	RCT	A maternity teaching hospital in Damascus, Syria	A total of 876 women were followed up in the three study groups: group A (285 women) 4 PNC visit; group B (294 women) 1 PNC visit; and group C (297 women) no visit (control)	577/296 for NMR and 498/258 for EBF outcome	Maternal postpartum morbidities; postnatal care uptake; contraceptive uptake and type; infant morbidities; infant immunization at three months; and exclusive breastfeeding during the first four months of life.	
Bhandari 2012 [44]	Cluster RCT	The trial was conducted in communities with a population of 1.1 million served by 18 primary health centers in the district of Faridabad, Haryana, India	29,667 and 30,813 newborns in intervention and control clusters, respectively	29,667/30,813 for NMR and 6204/6163 for EBF outcome	Neonatal and infant mortality; newborn care practices, exclusive breastfeeding at 4 weeks	
Coutinho 2005 [37]	RCT	Urban areas of Palmares and three neighboring small towns (Catende, Água Preta, and Joaquim Nabuco) in the interior of the State of Pernambuco, northeastern Brazil). The area is hilly and lies 130 km southwest of Recife, the State capital.	175 control and 175 intervention mother and their newborn/infants	175/175	Rates of exclusive breastfeeding at 12 weeks of age over 24 h recall and breastfeeding practices	Compared the hospital-based intervention with a combined hospital-based and community-based
Darmstadt 2010 [45]	Cluster RCT	The trial was implemented in Mirzapur, a sub-district of Tangail district, Dhaka Division, Bangladesh, located 2 h by car from the capital city of Dhaka	4616 and 5241 live births were recorded from 9987 and 11,153 participants in the comparison and intervention arm	301/265	Antenatal and immediate newborn care behaviors, knowledge of danger signs, care-seeking for neonatal complications, and neonatal mortality.	
Kirkwood 2013 [29]	Cluster RCT	Undertaken in seven predominantly rural districts in the Brong Ahafo Region, Ghana: Kintampo North, Kintampo South, Nkoranza North, Nkoranza South, Tain, Techiman, and Wench	11,419 and 11,144 newborn and mothers in intervention and control groups, respectively	11,419/11,144 for NMR and 1414/1371 for EBF outcome	Neonatal mortality rate and coverage of key essential newborn-care practices; exclusive breastfeeding in the previous 24 h between days 26 to 32 after birth	Same study area with “Pitt 2016”
Kumar 2008 [27]	Cluster RCT	Shivgarh, a rural block in Uttar Pradesh, with a population of 104,123. Socioeconomic indicators are among the lowest in the state.	1522 intervention and 1079 control groups of mothers and newborn in Shivgarh	1522/1079	Changes in newborn care practices and neonatal mortality rate compared with the control group	
LeFevre 2013 [40]	Cluster RCT	Beanibazar, Zakiganj and Kanaighat subdistricts of Sylhet division; a division which has a higher level of neonatal mortality and a higher fertility rate than any of the other five of Bangladesh’s divisions	364 and 750 mothers and newborns in intervention and control groups, respectively	364/750	Cost-effectiveness of two strategies (home and community care) for neonatal and maternal care	Neonatal mortality is reported in “Baqui 2016”.
Memon 2015 [41]	quasi-experimental	Gilgit district which is situated about 600 km away from Islamabad, the capital of Pakistan. The population of district Gilgit is around 283,324, the majority of which are subsistence farmers. The health infrastructure comprised of three Basic Health Units, one Rural Health Centre, five Civil Hospitals and one District Head Quarter Hospital.	833 and 842 mothers and newborns in the intervention and control groups, respectively, in a remote mountainous district in Northern Pakistan	458/463	Changes in maternal and newborn care practices and perinatal and neonatal mortality rates	
Pitt 2016 [43]	Cluster RCT	Undertaken in seven predominantly rural districts in the Brong Ahafo Region, Ghana: Kintampo North, Kintampo South, Nkoranza North, Nkoranza South, Tain, Techiman, and Wench	11,419 mothers and their newborns in intervention and 11,144 Mothers and their newborns in control groups in seven districts of rural Ghana	11,419/11,144	Cost-effectiveness of home visits to women and their newborns for interventions and cost per newborn saved life.	Neonatal mortality is reported in “Kirkwood 2013”
Soofi 2017 [42]	Cluster RCT	Naushahro Feroze district of rural Sindh. The district is located 450 km north of Karachi with a population of around 1·3 million	736 mothers and newborns in intervention and 1050 mothers and newborns in control groups	736/1050	All-cause neonatal mortality	
Tyllerskr 2011 [36]	Cluster RCT	The study was undertaken in rural Banfora, southwest Burkina Faso, Mbale District, eastern Uganda, and Paarl (a periurban site close to Cape Town), Umlazi (periurban site near Durban), and Rietvlei (rural Kwa Zulu Natal), South Africa.	2579 mother-infant pairs to the intervention or control clusters	1323/1256	Prevalence of exclusive breastfeeding and diarrhea reported by mothers regarding infants aged 24 weeks over 24 h recall	
Waiswa 2015 [47]	Cluster RCT	Iganga and Mayuge districts in eastern Uganda, within the Iganga-Mayuge Health and Demographic Surveillance Site (HDSS). The HDSS is predominately rural, comprising 65 villages and a total population of approximately 70,000.	894 and 893 mothers and newborns in the intervention and control groups, respectively	894/893	Coverage of key essential newborn care behaviors (breastfeeding, thermal care, and cord care); exclusive breastfeeding over neonatal period	Health facility strengthening was done to improve the quality of care in all intervention and control sites

Most of the trials were conducted in predominantly rural districts that had poor infrastructure (poor roads, communications, education, and health services). However, three studies were conducted in urban [33] or peri-urban [36, 37] settings which were within a short distance to the countries’ capital cities (Table 1).

Twelve trials recruited CHWs and three studies employed health professionals including midwives and nurses. Health workers were trained for about a week to make home visits to offer preventive and promotive newborn care services. Table 2 presents the intervention packages implemented in each trial. All trials conducted postpartum home visits to promote newborn care practices; 11 trials did both antepartum and postpartum home visits. Moreover, nine trials conducted community activities [28, 29, 38–43] in addition to home visits to promote newborn care practices. Five of the studies trained providers/CHWs to identify sick newborns, refer them to facilities, and if referral was not possible to treat sick newborns in addition to preventive and promotive newborn care services [38, 40, 42, 44, 45] (Table 2).

**Table 2:** Descriptions of interventions

Study ID	Interventions	Type of care	Intervention providers and training	Number of ANC visits	Number of PNC visits	Timing of PNC visits	Length of postpartum follow-up (in weeks)	Postpartum visit coverage (%)	Comparator type
Bang 1999 [38]	Village women health workers were recruited and trained to provide health education to mothers and treat sick neonates	Home visit and community activities to promote optimal neonatal care practices and treatment of sick neonates	Women village health workers (5–10 grade)	1	8	days 1, 2, 3, 5, 7, 14, 21, 28 & on any other day if the family called	4	84	Routine prenatal care, immunization, family planning, control of communicable diseases, and curative medical care were provided in the government facilities
Baqui 2016 [39]	Both home care and community care maternal and neonatal health service delivery strategies by Community Health Workers (CHWs); community mobilization and behavior-change communication to promote birth and newborn-care practice	Home visit and community activities to promote optimal neonatal care practices	CHWs trained for 5 days	2	3	days 1, 3 & 7	1	79	Active facility-based comparator
Bashour 2008 [33]	Home visits to examine and counsel women	Home visit to promote optimal neonatal care practices	Midwives trained for 5 days	0	5	Women in group A received 4 home visits on days 1, 3, 7, & 30 women in group B received 1 home visit on day 3	4	100	Usual hospital care without home visit in the postpartum period
Bhandari 2012 [44]	Postnatal home visits to promote breastfeeding, delaying bathing, keeping the baby warm, cord care, care-seeking for illness and treated sick newborns and older children	Home visit to promote optimal neonatal care practices and treatment of sick neonates	CHWs, nurses, and physicians trained for 11 days	0	3	days 1, 3, & 7	6	90	Usual or routine facility-based care
Coutinho 2005 [37]	Home visits by women with secondary school education to promote and support exclusive breastfeeding	Promote optimal neonatal care practices	Health care providers, midwives and nursing assistants trained for 20 h	0	10	days 3, 7, 15, & 30 and every 2 weeks during the second month, and once a month during the 3–6 months	24	83	Women’s usual stay facility 24 h to 48 h after deliveries. Maternity staff would counsel and encourage mothers to initiate and maintain exclusive breastfeeding
Darmstadt 2010 [45]	-Pregnancy surveillance to identify pregnancies by CHW -Antepartum home visits to promote birth and newborn care preparedness -Postpartum home visits to promote preventive care practices and to assess newborns for illness, and referred sick neonates	Home visit to promote optimal neonatal care practices and treatment of sick neonates	CHWs trained for 36 days on pregnancy surveillance, negotiation skills, essential newborn care, neonatal illness surveillance and management of illness	2	3	days 1, 2, 5 & 8	1	69	Usual health services provided by the government, non-governmental organizations and private providers
Kirkwood 2013 [29]	Community-based surveillance volunteers were trained to identify pregnant women and to make home visits during pregnancy and postpartum to promote essential newborn-care practices, weigh and assess babies for danger signs, and refer as necessary; community-wide meetings	Home visit and community activities to promote optimal neonatal care practices	community-based surveillance volunteers (CBSVs) (trained for 9 days)	2	3	days 1, 3, & 7	1	63	Routine maternal and child health care (ANC, facility delivery, postpartum check-ups, infant welfare).
Kumar 2008 [28]	CHWs provided preventive & promotive package of interventions for essential newborn care (birth preparedness, clean delivery, and cord care, thermal care, breastfeeding promotion, and danger sign recognition); community-based intervention for behavior change management	Home visit and community activities to promote optimal neonatal care practices	CHWs	2	2	days 1 & 3	1	68	Usual services (ANC, delivery, PNC, and vaccination services)
LeFevre 2013 [40]	-CHWs provided an initial dose of antibiotic treatment to the infant with suspected severe neonatal illness and to promote the referral -Community mobilization and behavior-change communication to promote birth and newborn-care preparedness	Home visit and community activities to promote optimal neonatal care practices and treatment of sick neonates	CHWs (secondary school education) trained for 5 days	2	3	days 1, 3 & 7	1	79	Pre-existing level of care
Memon 2015 [41]	Promotion of ANC, nutrition, skilled delivery, and healthy newborn care practices; community mobilization and awareness creation	Home visit and community activities to promote optimal neonatal care practices	Lay health workers (LHW)/CHWs; LHW, local resident women with 8 grade of formal education were trained for 18 months	2	1	Monthly	1	83	Routine health services
Pitt 2016 [43]	Antepartum and postpartum home visits to promote essential newborn-care practices and assess babies for danger signs, and refer as necessary); facilitated community-wide meetings	Home visit and community activities to promote optimal neonatal care practices	Community volunteers	2	3	days 1, 3, & 7	1	63	Routine maternal and child health care (ANC, facility delivery, postpartum check-ups, infant welfare)
Soofi 2017 [42]	Lady Health Workers (LHW) provided community mobilization and education package and recognition of possibly asphyxiated newborn infants at birth and bag and mask resuscitation as needed, and recognition and management of suspected neonatal infections.	Home visit and community activities to promote optimal neonatal care practices and treatment of sick neonates	LHW received an initial 3 days of training and monthly 1-day refresher sessions thereafter	0	4	attend deliveries and days 3, 7, 14, & 28 after birth	4	30	-LHW program continued to function as usual. -They continued to have regular monthly debriefing and refresher training according to the standard national LHW program
Tyllerskr 2011 [36]	Trained peer counselors made antenatal and postpartum breastfeeding peer counseling visits	Home visit to promote optimal neonatal care practices	Peer counselors trained for 1 week	1	4	-In Burkina Faso: home visits at weeks 1, 2, 4, 8, 16, and 20 -In Uganda and South Africa: home visits at weeks 1, 4, 7, and 10	6	100	-Standard health care only in Burkina Faso and Uganda -Home visit by peer counselors in South Africa, with the same schedule as in the intervention clusters, but assisted families in obtaining birth certificates and social welfare grants
Waiswa 2015 [47]	Villages volunteer CHWs were trained to identify pregnant women and make home visits to offer preventive and promotive care and counseling, with extra visits for sick and small newborns to assess and refer	Home visit to promote optimal neonatal care practices	CHWs trained for 5 days	2	3	first week after birth	1	63	Standard health services, in addition to the improved health facilities

Regarding the implementation strength of the studies, defined here as frequency of home visits and coverage of visits, each trial had an average of 4 postpartum home visits and an average of 1 antepartum visit (usually at third trimester). Likewise, coverage of home visits, proportion of mothers and/or their newborns received postpartum home visit, ranged from 30 to 100% (median 79%).

Studies’ follow-up period ranged from 1 to 24 weeks postpartum and more than half of the studies (53%, *n* = 8) followed-up mothers and their newborns for one week after delivery. The exclusive breastfeeding outcome definition varied across studies; three trials [29, 37, 44] determined exclusive breastfeeding at neonatal age while others determined at 3 months [36], 4 months [33], or 6 months [46]. Three studies observed exclusive breastfeeding in the previous 24 h recall at 12 weeks of age [37], 24 weeks of age [36], and age between days 26 to 32 after birth [29] (Table 2).

### Risk of bias in included studies

**Fig. 2:**
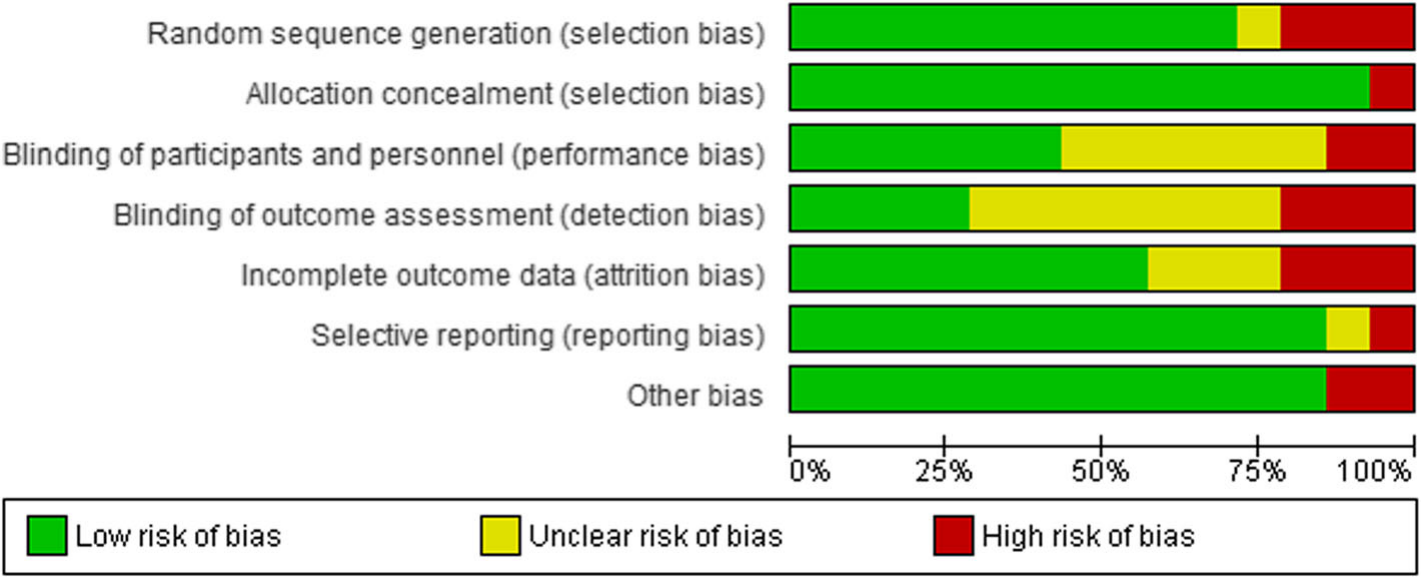
Risk of bias graph: review authors’ judgments about each risk of bias item presented as percentages across all included studies

The risk of bias of included trials is presented in Fig. 2.

### Allocation Bias

Most trials (*n* = 11; % = 79) included had low risk of random sequence generation bias [28, 29, 36, 37, 39, 40, 42–45, 47]; three studies had high risk of random sequence generation bias [33, 38, 41] and one trial had unclear risk of random sequence generation bias [47]. Except for one [41] all other studies had low risk of allocation concealment bias.

### Blinding

Blinding of participant and personnel was adequate in 11 of the studies included [27, 28, 33, 36, 38, 41–43, 45, 47, 48]. Four studies had high risk, either performance or detection bias [33, 37, 41, 42] and six and seven studies had an unclear risk of performance bias [29, 39–41, 43, 44] and detection bias [29, 38–40, 43, 44, 47], respectively.

### Incomplete outcome data and selective reporting

One trial had high risk of reporting bias [33]. Most studies had low risk of attrition; however, one trial [37] had unclear risk of reporting bias.

### Other potential sources of Bias

Other potential sources of bias including baseline imbalances were identified in two studies [33, 44]. Moreover, possible cluster-level time-varying unmeasured confounders including regional pregnancy leave and employment policy might bias the exclusive breastfeeding outcome. However, there is no compelling reason to believe that these systematically influenced the intervention areas but not in the comparison areas unless otherwise, it happened after allocation of the treatment.

### Effects of interventions

The Summary of Findings table gives estimates of the effects of home-based PNC on neonatal health outcomes as well as grading the level of evidence (Table 3).

**Table 3:** Home-based postnatal care compared to routine PNC for newborn health

Outcomes	Anticipated absolute effects (95% CI)		Relative effect(95% CI)	№ of participants (studies)	Certainty of the evidence(GRADE)
	Risk with routine PNC	Risk with home-based PNC			
Neonatal mortality	42 per 1000	32 per 1000 (26–39)	RR 0.76 (0.62–0.92)	93,083 (9 RCTs)	⨁⨁⨁◯ MODERATE
Exclusive breastfeeding	424 per 1000	680 per 1000 (536–796)	OR 2.88 (1.57–5.29)	20,624 (6 RCTs)	⨁⨁⨁⨁ HIGH

### Neonatal mortality

**Fig. 3:**
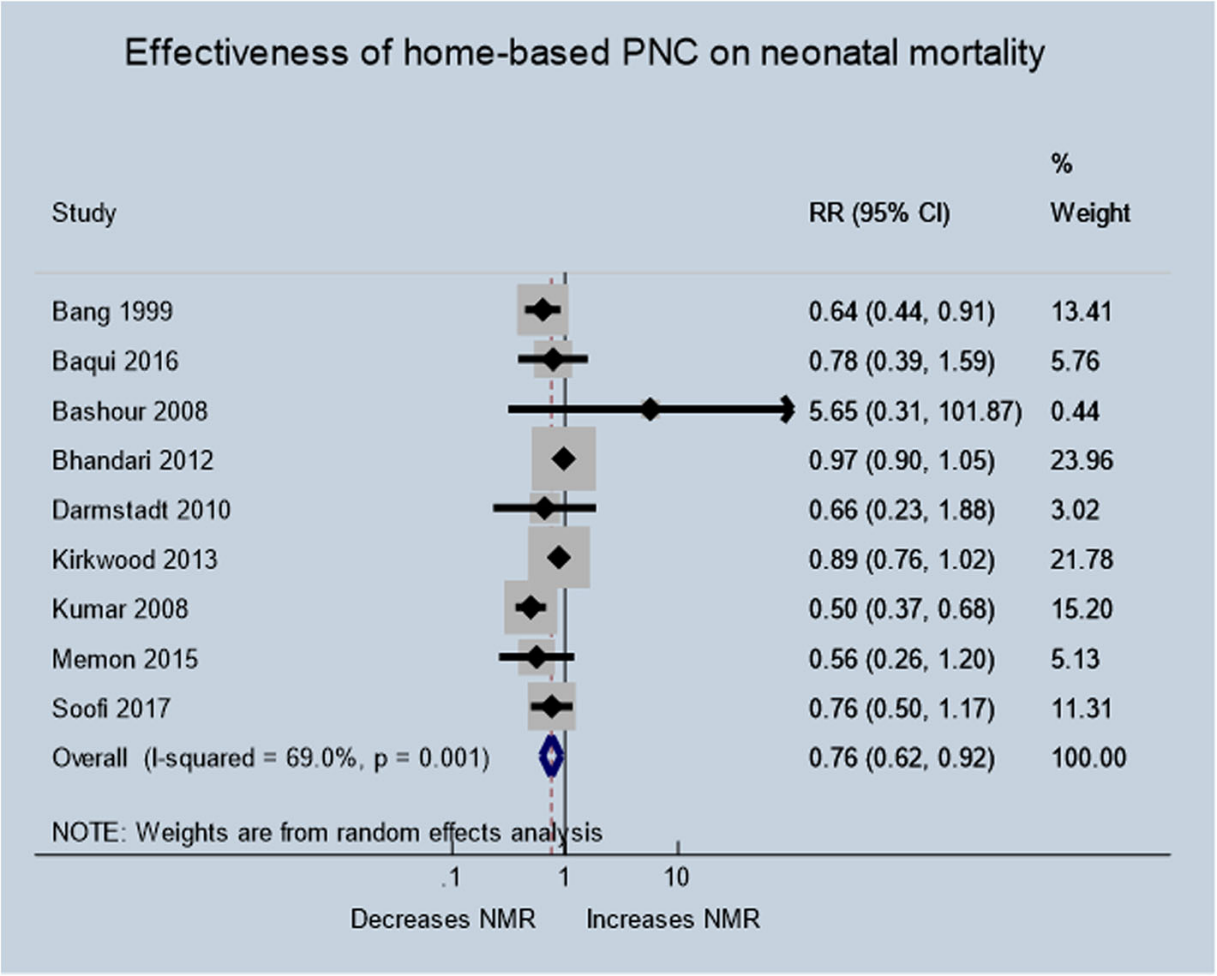
Effectiveness of home-based postnatal care on neonatal mortality

Nine studies [28, 29, 33, 38, 39, 41, 42, 44, 45] involving 93,083 participants reported the effect of home visits on neonatal mortality as compared with the routine postpartum care. As presented in Fig. 3 below, the pooled analysis showed that home-based PNC reduced neonatal mortality by 24% (RR 0.76, 95% CI 0.62–0.92) with substantial heterogeneity between studies (I^2^ = 69.0%; *p* = < 0.01; Tau^2^ = 0.0393).

### Sensitivity analysis

Sensitivity analysis was conducted excluding trials that evaluated the effect of home-based follow-up of neonates born in and recruited from hospitals [33]. Following removal of this trial, the overall pooled estimate increased by only 1% (RR 0.75, 95% CI 0.62–0.91) with a slight increase in heterogeneity between studies (I^2^ = 70.9%; *p* = .001) indicating no or little difference in the outcome and results were not driven by a trial that recruited newborns from hospitals.

### Subgroup analysis and meta-regression

The subgroup analysis revealed that more than three PNC home visits contributed to reduction in neonatal mortality (RR: 0.70; 95% CI: 0.53–0.91) than trials with less than three PNC home visits (RR: 0.77; 95% CI: 0.61–0.98; heterogeneity *p* = .043). Home visits by community health workers were associated with better survival of neonates (RR: 0.69; 95% CI: 0.55–0.87) than visits by health professionals (RR: 1.26; 95% CI: 0.37–4.30; heterogeneity *P* = .001). Regarding intervention characteristics, community mobilization efforts with home visits to promote newborn care practices helped reduced neonatal mortality (RR: 0.69; 95% CI: 0.54–0.88) than home visits alone (RR: 0.97; 95% CI: 0.90–1.05; heterogeneity *P* = .001). Moreover, trials with publication year 2008 and before revealed greater reduction in neonatal mortality (RR: 0.58; 95% CI: 0.40–0.85) than publication year after 2008 (RR: 0.94; 95% CI: 0.88–1.01; heterogeneity *P* < .01).

Trials with coverage of home visits of 70% and above, showed non-statistically significant greater reduction in neonatal mortality (RR: 0.70; 95% CI: 0.50–0.99) than trials with less than 70% coverage of home visits (RR: 0.87; 95% CI: 0.76–1.00). Likewise, statistically non-significant trend towards a greater effect on mortality was observed with curative (inject-able antibiotics) and preventive interventions (RR: 0.82; 95% CI: 0.63–1.05), as compared to only preventive intervention (RR: 0.70; 95% CI: 0.48–1.03; heterogeneity *P* = .016) (Table 4).

**Table 4:** Subgroup analysis for neonatal mortality outcome

Predictor variables	# of trials	Random-effects model		Fixed-effects model		Test for heterogeneity		*p*-value for subgroup heterogeneity
		RR	95% CI	RR	95% CI	I^2^ (%)	*p*-value	
Overall	9	0.76	0.62–0.92	0.91	0.85–0.97	69.0	< 0.01	NA
Number of PNC visits								
> 3	4	0.70	0.53–0.91	0.70	0.53–0.91	0.0	0.485	0.043
< = 3	5	0.77	0.61–0.98	0.92	0.86–0.98	79.2	< 0.01	
Home visit coverage								
More than 70%	7	0.70	0.50–0.99	0.92	0.85–0.99	76.0	< 0.01	0.511
Less than 70%	2	0.87	0.76–1.00	0.87	0.76–1.00	0.0	0.517	
Type of provider								
Healthcare provider	2	1.26	0.37–4.30	0.98	0.90–1.05	29.5	0.234	0.001
CHW	7	0.69	0.55–0.87	0.77	0.68–0.86	52.8	0.048	
Intervention components								
Community mobilization & home visits	6	0.69	0.54–0.88	0.77	0.69–0.86	60.5	0.027	0.001
Home visits only	3	0.97	0.90–1.05	0.97	0.90–1.05	0.0	0.377	
Type of care								
Preventive	4	0.70	0.48–1.03	0.79	0.70–0.90	0.70	0.010	0.016
Preventive & curative	5	0.82	0.63–1.05	0.95	0.88–1.02	53.5	0.091	
Home visits								
Antepartum & postpartum	6	0.67	0.51–0.88	0.77	0.68–0.87	60.7	0.026	0.001
Postpartum only	3	0.93	0.74–1.17	0.97	0.90–1.04	25.0	0.264	
Publication year								
< =2008	3	0.58	0.40–0.85	0.56	0.45–0.71	43.9	0.168	< 0.01
> 2008	6	0.94	0.88–1.01	0.94	0.88–1.01	0.0	0.430	

In single-level meta-regression, only year of publication was significantly associated with neonatal mortality (*p* = <.01). However, in multiple level meta-regression, no independent predictor variables were found (result not shown).

### Test of publication bias

**Fig. 4:**
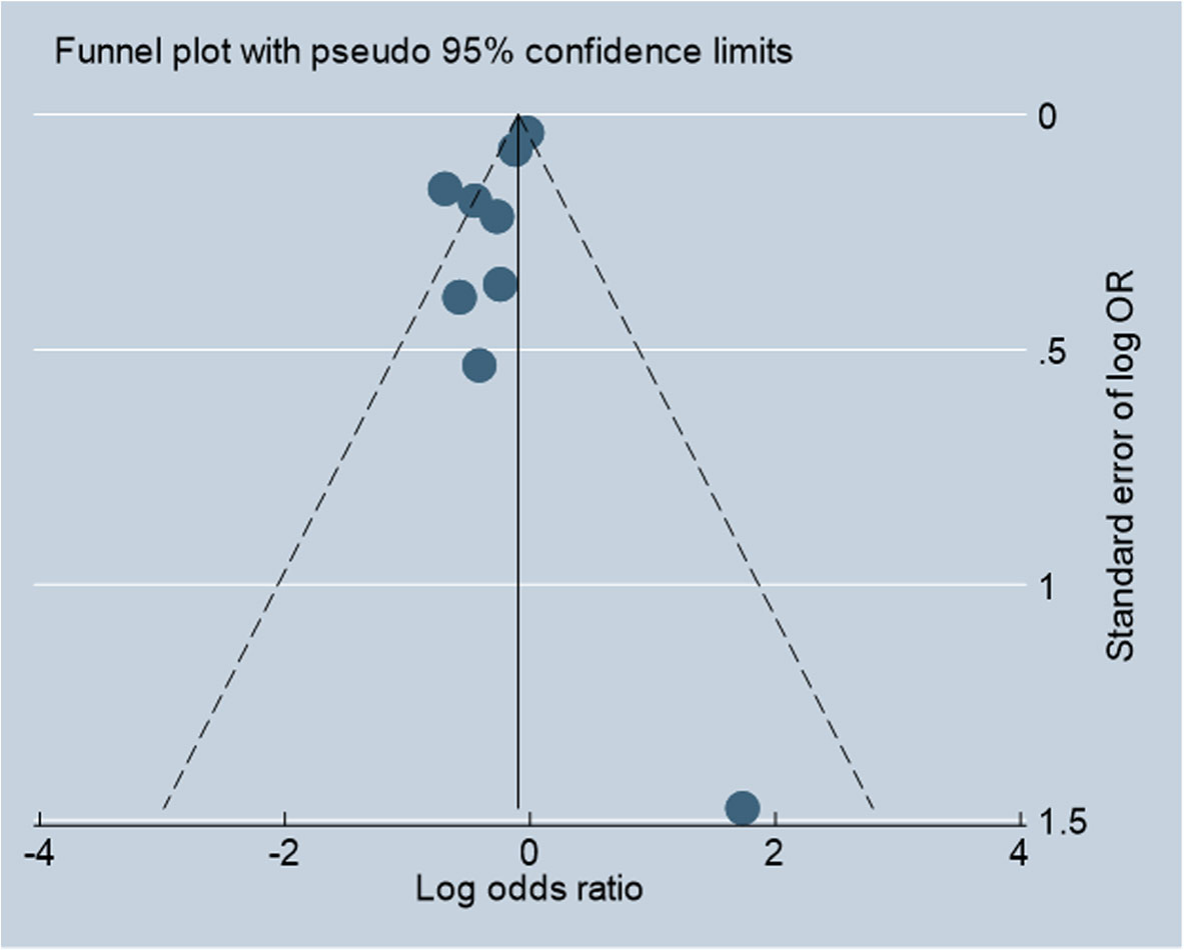
Funnel plot for neonatal mortality outcome

The funnel plot (Fig. 4) appeared symmetrical, which suggests no evidence of small-study effects. The Egger’s test also indicated low possibility of publication bias (Coef. = − 1.263; *p* = .130).

### Exclusive breastfeeding

**Fig. 5:**
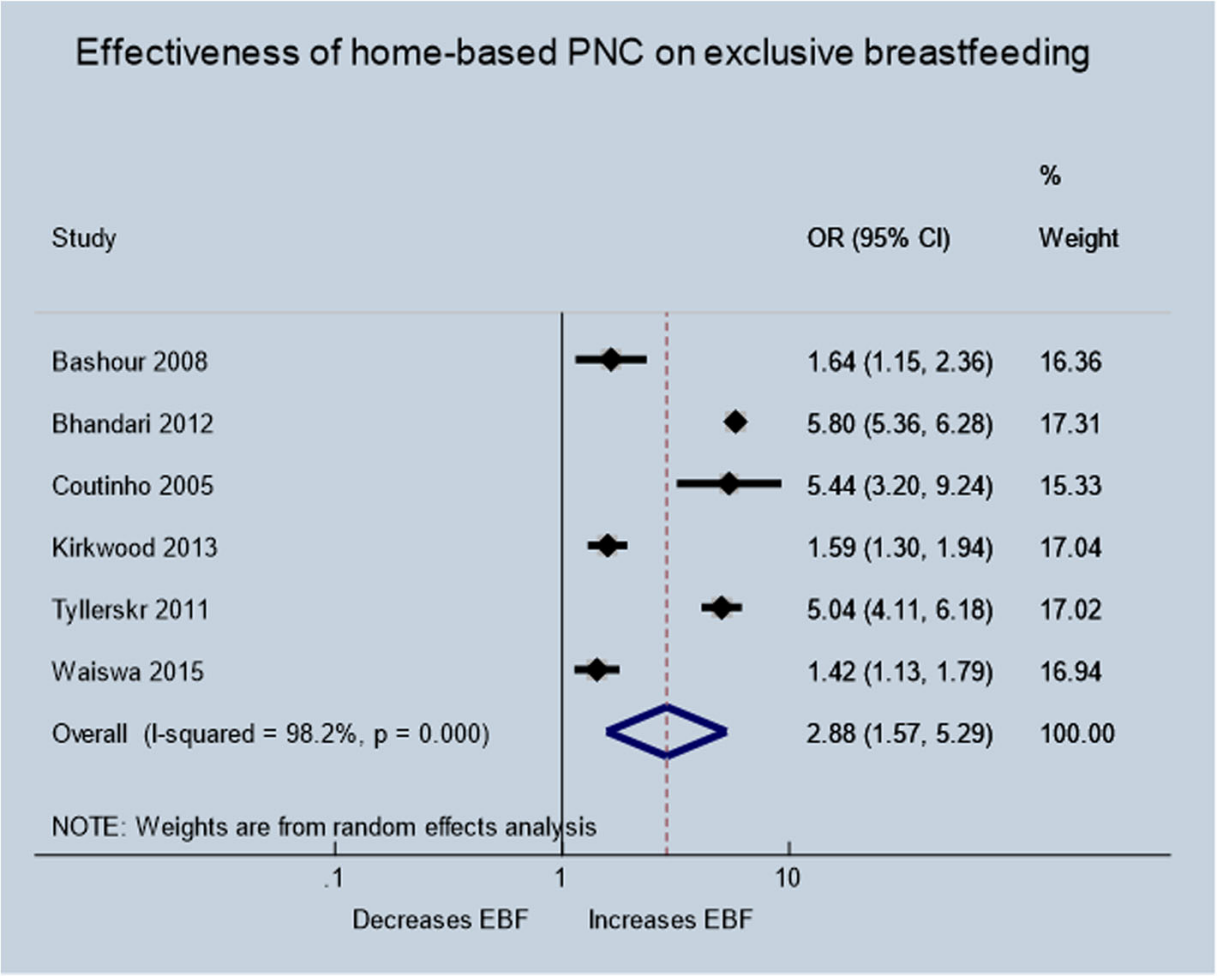
Effectiveness of home-based postnatal care on exclusive breastfeeding practice

Six trials [29, 33, 36, 37, 44, 47] involving 20,624 mothers reported on the exclusive breastfeeding outcome. The pooled analysis showed (Fig. 5) that the odds of exclusive breastfeeding practice of mothers in the home visit group were about three times higher than the routine care group [OR: 2.88; 95% CI:1.57–5.29) with substantial heterogeneity between studies (I^2^ = 98.2%; *p* = <.01; Tau^2^ = 0.5517).

### Subgroup analysis

This outcome had substantial heterogeneity; as such, subgroup analysis was carried out. The subgroup analysis showed that groups of mothers who had more than 70% home visits (OR: 4.06; 95% CI: 2.60–6.36) were exclusively breastfeeding significantly more than their counterparts (OR: 1.51; 95% CI: 1.30–1.76). Likewise, groups of mothers who had more than four weeks of follow-up (OR: 5.69; 95% CI: 5.29–6.12) were exclusively breastfeeding significantly more than their counterparts (OR: 2.94; 95% CI: 0.91–9.50).

We found no significant difference among those mothers who had more than three visits (OR: 3.54; 95% CI: 1.64–7.65) and those who had less or equal to three home visits (OR: 2.36; 95% CI: 0.83–6.71). Though statistically non-significant, home visits by health professionals were associated with better exclusive breastfeeding practice (OR: 0.69; 95% CI: 2.60–8.69) than visits by community health workers (OR: 2.25; 95% CI: 1.01–5.29; heterogeneity *P* < .01). Trials with publication year 2008 and before did not show significant difference in exclusive breastfeeding practice (OR: 2.94; 95% CI: 0.91–9.50) than publication year after 2008 (OR: 2.86; 95% CI: 1.36–6.04; heterogeneity *P* < .01) (Table 5).

**Table 5:** Subgroup analysis in trials of home-based PNC visit on exclusive breastfeeding outcome

Predictor variables	No. of trials	Random-effects model		Fixed-effects model		Test for heterogeneity		*p*-value for subgroup heterogeneity
		OR	95% CI	OR	95% CI	I^2^ (%)	*p-*value	
Overall	6	2.88	1.57–5.29	4.29	4.02–4.57	98.2	< 0.01	NA
Number of PNC visits								
> 3	3	3.54	1.64–7.65	3.89	3.36–4.71	93.3	< 0.01	0.345
< = 3	3	2.36	0.83–6.71	4.34	4.05–4.66	99.2	< 0.01	
Home visit coverage								
> 70%	4	4.06	2.60–6.36	5.42	5.05–5.82	93.4	< 0.01	< 0.01
< = 70%	2	1.51	1.30–1.76	1.51	1.30–1.76	0.0	0.483	
Follow-up period								
> 4 weeks	3	5.69	5.29–6.12	5.69	5.29–6.12	0.0	< 0.01	< 0.01
< =4 weeks	3	1.53	1.33–1.76	1.53	1.33–1.76	0.0	< 0.01	
Age at exclusive breastfeeding								
1 month	3	3.65	1.37–9.76	4.88	4.54–5.25	98.6	< 0.01	< 0.01
> 1 month	3	2.29	0.4–5.56	2.64	2.30–3.04	97.3	< 0.01	
Type of provider								
Healthcare provider	3	3.73	2.60–8.69	5.47	5.07–5.91	95.6	< 0.01	< 0.01
CHW	3	2.25	1.01–5.29	2.31	2.05–2.61	97.7	< 0.01	
Publication year								
< =2008	2	2.94	0.91–9.50	2.40	1.78–3.23	92.5	< 0.01	< 0.01
> 2008	4	2.86	1.36–6.04	4.41	4.13–4.71	98.8	< 0.01	

### Test of publication bias

The funnel plot appeared symmetrical (result not shown). The Egger’s test also indicated low possibility of publication bias (Coef. = −7.870; *p* = .164).

### Cost-effectiveness

The cost-effectiveness results are presented in Table 6 below. Three trials reported the incremental cost for neonatal mortality outcomes. Different cost-effectiveness and cost-utility measures were used. All three trials reported cost per neonatal death averted [40, 38, 43] or newborn life-year saved [40, 43]. One trial used cost-utility measures (i.e., cost per DALYs gained) [40].

**Table 6:** Cost-effectiveness results

Study	Country	Intervention	Analytic view point	Quality	Cost-effectiveness measure	Cost-effectiveness result (US$ 2016)	GDP per capita	Neonatal Mortality Rate	
								Protective Effectiveness (%)	Control group
Bang 1999 [38]	India	Home-based neonatal care by village health workers	Program	Low	per neonatal death averted	13.86	1940	36.0	58.6
Pitt 2016 [43]	Ghana	Home visits made to pregnant women and their babies in the first week of life by community-based surveillance volunteers	Provider perspective	High	per discounted life-year saved	319	1641	11.0	32.8
LeFevre 2013 [40]	Bangladesh	Home visit made by community health workers to offer MNH services including postnatal home visits	Societal perspective (included program, provider and household costs)	High	Cost per neonatal death averted	2939	1517	22.0	43.7
					Cost per DALY averted	103.44			

According to World Health Organization’s Choosing Interventions that are Cost-Effective project (WHO-CHOICE) recommendations [49], considering cost per neonatal death averted, newborn life-year saved, and DALY averted measures, home-based neonatal care strategies were found to be cost-effective.

## Discussions

The objective of this study was to systematically review the existing body of knowledge that explores the effectiveness and cost-effectiveness of home-based postpartum care on neonatal mortality and exclusive breastfeeding practice in LMICs.

The pooled analysis showed that home-based postpartum care is effective in reducing neonatal mortality and promoting exclusive breastfeeding practice in LMICs which have poor access to health care. Though on meta-regression, no variable emerged as a significant predictor of an effect on neonatal mortality; the subgroup analysis suggested that more frequent postpartum home visits, combined community mobilization efforts with home visits, and interventions implemented prior to 2008 publication year, to had a greater reduction in neonatal mortality than their counterparts. Likewise, higher home visits may have encouraged more women to exclusively breastfeed.

The trials included in this review implemented a package of interventions that varied in intensity, components of interventions, duration of follow-up, and intervention provider. Some trials implemented both antepartum and postpartum home visits and other trials implemented postpartum home visits alone. Others included community mobilization efforts in addition to home-based promotion of newborn care practices. There were trials that included curative and preventive interventions, while others implemented preventive newborn care practices alone. Moreover, for breastfeeding outcome, there were variations in settings—some trials were conducted in predominantly in rural and others in peri-urban settings— as well as variations in how outcomes were measured and when—some trials determined exclusive breastfeeding at neonatal age while others determined at 3, 4, or 6 months. As such, due to the complex nature of the interventions, it was difficult to differentiate the independent effects of different components of interventions on neonatal mortality.

Our findings augment the WHO recommendations of home-based postpartum care strategy [19]. The findings are also in line with previous systematic reviews that report home-visit strategy as effective in reducing neonatal mortality [11]; cost-effective [15]; and promote the practice of exclusive breastfeeding [50].

Most of the trials were of acceptable quality with low or unknown risk of bias, except for the two trials where the possibility of bias was high—one trial [33] which used block randomization based on estimated number of hospital deliveries per day and had significant baseline imbalances and the other trial [41] which was quasi-experimental. We graded the quality of the evidence using the GRADE approach; the evidence was graded as moderate for neonatal mortality outcome (Table 3, above), with downgrading decisions due to risks of bias and imprecision of effects, and high for exclusive breastfeeding.

This review provides several lessons for implementers of MNH programs in LMICs like Ethiopia. First, lower-and middle-income countries have shortage of trained health care providers [51–53] as well as inequitable distribution of providers [54, 55] which makes it difficult to intensify MNH care practices at facility level. This review illustrates that home-based MNH care can be practiced by community members through short term training on the packages needed to implement. Second, providing home-based MNH care might enable the implementers to access hard-to-reach areas, because people in LMICs have lower health-seeking behavior and they are far from health facilities.

In this review, we incorporated relevant subgroup and meta-regression analyses, explored any unit of analysis errors in the cluster randomized trials and appropriately combined all cluster- and individual-randomized trials together and no evidence of publication bias was found.

However, the review has limitations. First, there are high levels of unexplained heterogeneity. Second, in many countries, postpartum care is not clearly defined in national guidelines and standards [9]. This might create variability in the control group defining facility-level routine postpartum care. Third, the estimates of cost and resource used which are sensitive to variability across settings which might limit the generalizability and transferability of cost-effectiveness results beyond the study settings. Fourth, included studies used different cost-effectiveness and cost-utility measures as well as different analytic viewpoints which makes estimating the pooled estimate difficult. Finally, comparing cost-effectiveness outcomes against GDP per capita as a threshold does not necessarily ensure that the strategy is affordable.

Well-designed evaluation of such interventions is required to establish the effectiveness of different intervention packages as well as to formulate the optimal packages and optimal timing of home visits and specific responsibilities of community health workers. Analytic viewpoint is one dimension that can affect study results. Large multi-center economic evaluation studies examining the impact of different analytic perspectives, implementation strength and willingness-to-pay parameters both in the intervention and comparison groups are required to understand well the degree to which strategies can be replicated elsewhere.

## Conclusions

Home visits and community mobilization activities to promote optimal neonatal care practices by community health workers is associated with reduced neonatal mortality, increased practice of exclusive breastfeeding, and cost-effectiveness in improving newborn health outcomes for low-and-middle-income countries which have poor access to facility-based care. The subgroup analysis suggested that more than three PNC home visits, home visits by community health workers, community mobilization efforts with home visits, and interventions implemented prior to 2008 publication year to had better neonatal survival. Likewise, groups of mothers who received more home visit coverage and more weeks of follow-up were more exclusively breastfed their babies. The pooled estimates of outcome variables have high levels of unexplained heterogeneity so that results should be interpreted with caution. Furthermore, a well-designed evaluation study is warranted to formulate the optimal package of interventions for specific health cadres and optimal timing of home visits.

## Supplementary information

**Additional file 1.** Search Strategy.docx. This is a survey strategy used to search for articles from databases.

## Data Availability

The datasets used and/or analyzed during the current study are available from the corresponding author on reasonable request.

## Abbreviations

CHW: Community health worker
CI: Confidence interval
DALY: Disability-adjusted-life-years
GDP: Gross domestic product
GRADE: Grading of recommendations assessment, development and evaluation
ICC: Inter-class correlation coefficient
ISPOR: International society for pharmacoeconomics and outcome research
LMICs: Low-and-middle-income countries
MNH: Maternal and newborn health
NHS: National health service
OR: Odds ratio
PICO: Population, intervention, comparison, and outcome
PNC: Postnatal care
PRISMA: Preferred reporting items for systematic review and meta-Analysis
PROSPERO: International prospective register of systematic reviews
RCT: Randomized clinical trial
RevMan: Review manager
RR: Risk ratio
USD: US dollars
WHO: World health organization

## References

[1] Lawn JE, Cousens S, Zupan J. 4 million neonatal deaths: when? Where? Why? Lancet 2005;365(9462):891–900. doi:https://doi.org/10.1016/S0140-6736(05)71048-5. 10.1016/S0140-6736(05)71048-5 15752534

[2] Bhutta ZA, Das JK, Bahl R, Lawn JE, Salam RA, Paul VK et al. Can available interventions end preventable deaths in mothers, newborn babies, and stillbirths, and at what cost? Lancet 2014;384(9940):347–370. doi:https://doi.org/10.1016/S0140-6736(14)60792-3. 10.1016/S0140-6736(14)60792-3 24853604

[3] Adam T, Lim SS, Mehta S, Bhutta ZA, Fogstad H, Mathai M (2005). Cost effectiveness analysis of strategies for maternal and neonatal health in developing countries. Bmj..

[4] Lassi ZS, Mallick D, Das JK, Mal L, Salam RA, Bhutta ZA (2014). Essential interventions for child health. Reprod Health.

[5] WHO, UNICEF, UNFPA, World Bank Group, United Nations population division (2015). Trends in maternal mortality: 1990 to 2015 population and development review.

[6] Holmes W, Kennedy E. Reaching emergency obstetric care: overcoming the ‘second delay’. Melbourne, Burnet Institute on behalf of Compass; 2010.

[7] Darmstadt GL, Bhutta ZA, Cousens S, Adam T, Walker N, De Bernis L (2005). Evidence-based, cost-effective interventions: how many newborn babies can we save?. Lancet.

[8] Belachew T, Taye A, Belachew T. Postnatal care service utilization and associated factors among mothers in Lemo Woreda, Ethiopia. J Women’s Health Care. 2016;5(10.4172):2167–0420.1000318.

[9] Warren C, Daly P, Toure L, Mongi P. Postnatal care. Opportunities for Africa’s newborns Cape Town, South Africa: Partnership for Maternal, newborn and child. Health. 2006:79–90.

[10] Lain SJ, Roberts CL, Bowen JR, Nassar N (2015). Early discharge of infants and risk of readmission for jaundice. Pediatrics..

[11] Gogia S, Sachdev HS (2010). Home visits by community health workers to prevent neonatal deaths in developing countries: a systematic review. Bull World Health Organ.

[12] Lassi ZS (2015). Bhutta ZA.

[13] Marston C, Renedo A, McGowan C, Portela A (2013). Effects of community participation on improving uptake of skilled care for maternal and newborn health: a systematic review. PLoS One.

[14] Bath J, Wakerman J (2015). Impact of community participation in primary health care: what is the evidence?. Australian Journal of Primary Health.

[15] Prost A, Colbourn T, Seward N, Azad K, Coomarasamy A, Copas A et al. Women's groups practising participatory learning and action to improve maternal and newborn health in low-resource settings: a systematic review and meta-analysis. Lancet 2013;381(9879):1736–1746. doi:https://doi.org/10.1016/S0140-6736(13)60685-6. 10.1016/S0140-6736(13)60685-6 PMC3797417 23683640

[16] Bhutta ZA, Soofi S, Cousens S, Mohammad S, Memon ZA, Ali I et al. Improvement of perinatal and newborn care in rural Pakistan through community-based strategies: a cluster-randomised effectiveness trial. Lancet 2011;377(9763):403–412. doi:https://doi.org/10.1016/S0140-6736(10)62274-X. 10.1016/S0140-6736(10)62274-X 21239052

[17] Bhutta ZA, Darmstadt GL, Hasan BS, Haws RA (2005). Community-based interventions for improving perinatal and neonatal health outcomes in developing countries: a review of the evidence. Pediatr.

[18] Mangham-Jefferies L, Pitt C, Cousens S, Mills A, Schellenberg J (2014). Cost-effectiveness of strategies to improve the utilization and provision of maternal and newborn health care in low-income and lower-middle-income countries: a systematic review. BMC Pregnancy Childbirth..

[19] WHO (2014). WHO recommendations on Postnatal care of the mother and newborn.

[20] The World Bank. Official exchange rate (LCU per US$, period average). International Monetary Fund, International Financial Statistics. World Bank, http://data.worldbank.org/indicator/PA.NUS.FCRF. 2017. Accessed 07/23/2018 2018.

[21] The World Bank. GDP per capita (current US$):World Bank national accounts data, and OECD National Accounts data files. World Bank, https://data.worldbank.org/indicator/NY.GDP.PCAP.CD. 2017. Accessed 07/30/2018 2018.

[22] Mathewos B, Owen H, Sitrin D, Cousens S, Degefie T, Wall S (2017). Community-Based Interventions for Newborns in Ethiopia (COMBINE): Cost-effectiveness analysis. Health Policy Plann.

[23] Higgins JP (2011). Green S.

[24] Cochrane T (2008). Review manager (RevMan) 5.3.

[25] GRADEpro G. GRADEpro guideline development tool [software]. McMaster Univ. 2015;435.

[26] Ramsey S, Willke R, Briggs A, Brown R, Buxton M, Chawla A (2005). Good research practices for cost-effectiveness analysis alongside clinical trials: the ISPOR RCT-CEA task force report. Value Health.

[27] Kumar V, Kumar A, Das V, Srivastava NM, Baqui AH, Santosham M (2012). Community-driven impact of a newborn-focused behavioral intervention on maternal health in Shivgarh, India. Int J Gynecol Obstet.

[28] Kumar V, Mohanty S, Kumar A, Misra RP, Santosham M, Awasthi S (2008). Effect of community-based behaviour change management on neonatal mortality in Shivgarh, Uttar Pradesh, India: a cluster-randomised controlled trial. Lancet.

[29] Kirkwood BR, Manu A, ten Asbroek AH, Soremekun S, Weobong B, Gyan T (2013). Effect of the Newhints home-visits intervention on neonatal mortality rate and care practices in Ghana: a cluster randomised controlled trial. Lancet.

[30] Hanson C, Manzi F, Mkumbo E, Shirima K, Penfold S, Hill Z (2015). Effectiveness of a home-based Counselling strategy on neonatal care and survival: a cluster-randomised trial in six districts of rural southern Tanzania. PLoS Med.

[31] Nair N, Tripathy P, Sachdev HS, Pradhan H, Bhattacharyya S, Gope R (2017). Effect of participatory women's groups and counselling through home visits on children's linear growth in rural eastern India (CARING trial): a cluster-randomised controlled trial. Lancet Glob Health.

[32] Manandhar DS, Osrin D, Shrestha BP, Mesko N, Morrison J, Tumbahangphe KM (2004). Effect of a participatory intervention with women's groups on birth outcomes in Nepal: cluster-randomised controlled trial. Lancet.

[33] Bashour HN, Kharouf MH, AbdulSalam AA, El Asmar K, Tabbaa MA, Cheikha SA (2008). Effect of postnatal home visits on maternal/infant outcomes in Syria: a randomized controlled trial. Public Health Nurs.

[34] Sterne JA, Palmer TM. Meta-analysis in Stata: an updated collection from the Stata Journal. 2 ed. StataCorp LP; 2016.

[35] StataCorp S. Statistical software: release 15. 2017.

[36] Tylleskar T, Jackson D, Meda N, Engebretsen IMS, Chopra M, Diallo AH (2011). Exclusive breastfeeding promotion by peer counsellors in sub-Saharan Africa (PROMISE-EBF): a cluster-randomised trial. Lancet.

[37] Coutinho SB, de Lira PIC, de Carvalho LM, Ashworth A (2005). Comparison of the effect of two systems for the promotion of exclusive breastfeeding. Lancet.

[38] Bang AT, Bang RA, Baitule SB, Reddy MH, Deshmukh MD (1999). Effect of home-based neonatal care and management of sepsis on neonatal mortality: field trial in rural India. Lancet.

[39] Baqui A, Williams E, El-Arifeen S, Applegate J, Mannan I, Begum N (2016). Effect of community-based newborn care on cause-specific neonatal mortality in Sylhet district, Bangladesh: findings of a cluster-randomized controlled trial. J Perinatol.

[40] LeFevre AE, Shillcutt SD, Waters HR, Haider S, El Arifeen S, Mannan I (2013). Economic evaluation of neonatal care packages in a cluster-randomized controlled trial in Sylhet, Bangladesh. Bull World Health Organ.

[41] Memon ZA, Khan GN, Soofi SB, Baig IY, Bhutta ZA (2015). Impact of a community-based perinatal and newborn preventive care package on perinatal and neonatal mortality in a remote mountainous district in northern Pakistan. BMC Pregnancy Childbirth.

[42] Soofi S, Cousens S, Turab A, Wasan Y, Mohammed S, Ariff S (2017). Effect of provision of home-based curative health services by public sector health-care providers on neonatal survival: a community-based cluster-randomised trial in rural Pakistan. Lancet Glob Health.

[43] Pitt C, Tawiah T, Soremekun S, ten Asbroek AHA, Manu A, Tawiah-Agyemang C et al. Cost and cost-effectiveness of newborn home visits: findings from the Newhints cluster-randomised controlled trial in rural Ghana. Lancet Glob Health 2016;4(1):e45-e56. doi:https://doi.org/10.1016/S2214-109X(15)00207-7. 10.1016/S2214-109X(15)00207-7 PMC5357735 26639857

[44] Bhandari N, Mazumder S, Taneja S, Sommerfelt H, Strand TA (2012). Effect of implementation of Integrated Management of Neonatal and Childhood Illness (IMNCI) programme on neonatal and infant mortality: cluster randomised controlled trial. BMJ (Clinical research ed).

[45] Darmstadt GL, Choi Y, Arifeen SE, Bari S, Rahman SM, Mannan I (2010). Evaluation of a cluster-randomized controlled trial of a package of community-based maternal and newborn interventions in Mirzapur. Bangladesh PloS one.

[46] Waiswa P, Manzi F, Mbaruku G, Rowe A, Marx M, Tomson G (2017). Effects of the EQUIP quasi-experimental study testing a collaborative quality improvement approach for maternal and newborn health care in Tanzania and Uganda. Implement Sci.

[47] Waiswa P, Pariyo G, Kallander K, Akuze J, Namazzi G, Ekirapa-Kiracho E (2015). Effect of the Uganda newborn study on care-seeking and care practices: a cluster-randomised controlled trial. Glob Health Action.

[48] Fottrell E, Azad K, Kuddus A, Younes L, Shaha S, Nahar T (2013). The effect of increased coverage of participatory women’s groups on neonatal mortality in Bangladesh: a cluster randomized trial. JAMA Pediatr.

[49] Marseille E, Larson B, Kazi DS, Kahn JG, Rosen S (2014). Thresholds for the cost–effectiveness of interventions: alternative approaches. Bull World Health Organ.

[50] Yonemoto Naohiro, Dowswell Therese, Nagai Shuko, Mori Rintaro (2014). Schedules for home visits in the early postpartum period. Evidence-Based Child Health: A Cochrane Review Journal.

[51] Kerber KJ, de Graft-Johnson JE, Bhutta ZA, Okong P, Starrs A, Lawn JE (2007). Continuum of care for maternal, newborn, and child health: from slogan to service delivery. Lancet.

[52] Munabi-Babigumira S, Glenton C, Lewin S, Fretheim A, Nabudere H (2017). Factors that influence the provision of intrapartum and postnatal care by skilled birth attendants in low- and middle-income countries: a qualitative evidence synthesis. Cochrane Database Syst Rev.

[53] Task Force on Health Systems Research. Informed choices for attaining the millennium development goals: towards an international cooperative agenda for health-systems research. Lancet 2004;364(9438):997–1003. 10.1016/S0140-6736(04)17026-8 15364193

[54] Gerein N, Green A, Pearson S (2006). The implications of shortages of health professionals for maternal health in sub-Saharan Africa. Reproductive Health Matters.

[55] Ahmed SM, Hossain MA, RajaChowdhury AM, Bhuiya AU (2011). The health workforce crisis in Bangladesh: shortage, inappropriate skill-mix and inequitable distribution. Hum Resour Health.

