# Supplementary material for: Effectiveness and cost-effectiveness of home-based postpartum care on neonatal mortality and exclusive breastfeeding practice in low-and-middle-income countries: a systematic review and meta-analysis
Source: BMC Pregnancy Childbirth. 2019 Dec 18;19:507. doi: 10.1186/s12884-019-2651-6 (PMC6921506; doi:10.1186/s12884-019-2651-6)
Supplement: Supplementary file 1 — Additional file 1. Search Strategy.docx. This is a survey strategy used to search for articles from databases. [file 12884_2019_2651_MOESM1_ESM.docx]

## Additional file 1: Search Strategy.

1. **Search Strategy PubMed and CENTRAL**

| Population | Intervention/Comparison | Outcome | Setting | Overall |
| --- | --- | --- | --- | --- |
| **#1** ((((((((((((postnatal care[MeSH Terms]) OR postpartum) OR puerper*) OR post partum) OR post natal) OR neonatal) OR newborn))) | **#2** ((((((((((((((((((((((home visit[MeSH] OR home care) OR facilit*) OR hospital care) OR health center care) OR facility based care) OR home care service*))))) | #3 (((((((((((((((((((((((cost benefit analysis [MeSH}] OR cost analysis) OR efficien*) OR health care cost) OR economic evaluation) OR cost effectiveness) OR impact OR effect*)))))))) OR ((((("infant mortality"[MeSH Terms] OR neonatal mortality OR death OR mortality OR perinatal death OR perinatal mortality OR stillbirth))) OR ((("breast feeding"[MeSH Terms] OR exclusive breastfeeding))))  #4 (#1 AND #2 AND #3) | **#5** (Africa OR Asia OR Caribbean OR West Indies OR South America OR Latin America OR Central America OR Middle East)  **#6** (Afghanistan OR Albania OR Armenia OR Armenian OR Bangladesh OR Benin OR Belize OR Bhutan OR Bolivia OR Burkina Faso OR Burkina Fasso OR Upper Volta OR Burundi OR Urundi OR Cambodia OR Khmer Republic OR Kampuchea OR Cameroon OR Cameroons OR Cameron OR Camerons OR Cameroun OR Cape Verde OR Central African Republic OR Chad OR Comoros OR Comoro Islands OR Mayotte OR Congo OR Zaire OR Cote d'Ivoire OR Ivory Coast OR Djibouti OR French Somaliland OR East Timor OR East Timur OR Timor Leste OR Egypt OR United Arab Republic OR El Salvador OR Eritrea OR Ethiopia OR Fiji OR Gambia OR Gaza OR Georgia OR Georgian OR Ghana OR Gold Coast OR Guatemala OR Guinea OR Guam OR Guiana OR Guyana OR Haiti OR Honduras OR India OR Indonesia OR Iraq OR Kenya OR Kiribati OR Korea OR Kosovo OR Kyrgyzstan OR Kirghizia OR Kyrgyz Republic OR Kirghiz OR Kirgizstan OR Lao PDR OR Laos OR Lesotho OR Basutoland OR Liberia)  **#7** (Madagascar OR Malagasy Republic OR Malawi OR Nyasaland OR Mali OR Marshall Islands OR Mauritania OR Agalega Islands OR Micronesia OR Moldova OR Moldovia OR Moldovian OR  Morocco OR Ifni OR Mozambique OR Myanmar OR Myanma OR Burma OR Nepal OR Netherlands Antilles OR New Caledonia OR Nicaragua OR Niger OR Nigeria OR Pakistan OR Palestine OR Paraguay OR Philippines OR Philipines OR Phillipines OR Phillippines OR Rwanda OR Ruanda OR Samoa OR Samoan Islands OR Navigator Island OR Navigator Islands OR Sao Tome OR Senegal OR Sierra Leone OR Sri Lanka OR Ceylon OR Solomon Islands OR Somalia OR Sudan OR Swaziland OR Syria OR Tajikistan OR Tadzhikistan OR Tadjikistan OR Tadzhik OR Tanzania OR Togo OR Togolese Republic OR Tonga OR Trinidad OR Tobago OR Tunisia OR Turkey OR Turkmenistan OR Turkmen OR Uganda OR Ukraine OR Uzbekistan OR Uzbek OR Vanuatu OR New Hebrides OR Vietnam OR Viet Nam OR West Bank OR Yemen OR Zambia OR Zimbabwe OR Rhodesia)  **#8** (developing countr* OR less developed countr * OR under developed countr * OR underdeveloped countr * OR middle income countr * OR low income countr* )  **#9** (low* GDP OR low* GNP OR low* gross domestic OR low* gross national)  **#10** (#5 OR #6 OR #7 OR #8 OR #9) | **#11** (#4 AND #10) AND (Clinical Trial[ptyp] AND "humans"[MeSH Terms] AND English[lang]) |

1. **Search strategy for Popline:**

(( ( ( home visit ) OR ( home care ) OR ( facilit* ) OR ( hospital care ) OR ( health center care ) OR ( facility based care ) OR ( home care service* ) ) ) AND ( ( ( postnatal care ) OR ( newborn care ) OR ( neonatal care ) ) ) AND ((( ( ( cost effectiveness ) OR ( cost benefit analysis ) OR ( cost analysis ) OR ( efficien* ) OR ( health care cost ) OR ( economic evaluation ) OR ( impact ) OR ( effect* ) (infant mortality) OR (breastfeeding))) ) )) AND ( ( Taxonomy term IDs from the <em class="placeholder">Language</em> vocabulary:English ) )
